# Effects of Moisture and Particle Size on Quantitative Determination of Total Organic Carbon (TOC) in Soils Using Near-Infrared Spectroscopy

**DOI:** 10.3390/s17102366

**Published:** 2017-10-17

**Authors:** Elena Tamburini, Fabio Vincenzi, Stefania Costa, Paolo Mantovi, Paola Pedrini, Giuseppe Castaldelli

**Affiliations:** 1 Department of Life Science and Biotechnology, University of Ferrara, Via L. Borsari, 46, 44121 Ferrara, Italy; fabio.vincenzi@unife.it (F.V.); stefania.costa@unife.it (S.C.); pdp@unife.it (P.P.); ctg@unife.it (G.C.); 2 Research Centre on Animal Production, CRPA, Viale Timavo, 43/2, 42121 Reggio Emilia, Italy; p.mantovi@crpa.it

**Keywords:** near infrared spectroscopy, soil, moisture effect, particle size effect, total organic carbon, spectra pretreatments

## Abstract

Near-Infrared Spectroscopy is a cost-effective and environmentally friendly technique that could represent an alternative to conventional soil analysis methods, including total organic carbon (TOC). Soil fertility and quality are usually measured by traditional methods that involve the use of hazardous and strong chemicals. The effects of physical soil characteristics, such as moisture content and particle size, on spectral signals could be of great interest in order to understand and optimize prediction capability and set up a robust and reliable calibration model, with the future perspective of being applied in the field. Spectra of 46 soil samples were collected. Soil samples were divided into three data sets: unprocessed, only dried and dried, ground and sieved, in order to evaluate the effects of moisture and particle size on spectral signals. Both separate and combined normalization methods including standard normal variate (SNV), multiplicative scatter correction (MSC) and normalization by closure (NCL), as well as smoothing using first and second derivatives (DV1 and DV2), were applied to a total of seven cases. Pretreatments for model optimization were designed and compared for each data set. The best combination of pretreatments was achieved by applying SNV and DV2 on partial least squares (PLS) modelling. There were no significant differences between the predictions using the three different data sets (*p* < 0.05). Finally, a unique database including all three data sets was built to include all the sources of sample variability that were tested and used for final prediction. External validation of TOC was carried out on 16 unknown soil samples to evaluate the predictive ability of the final combined calibration model. Hence, we demonstrate that sample preprocessing has minor influence on the quality of near infrared spectroscopy (NIR) predictions, laying the ground for a direct and fast in situ application of the method. Data can be acquired outside the laboratory since the method is simple and does not need more than a simple band ratio of the spectra.

## 1. Introduction

Soil is an essential pillar of agriculture and any form of human intervention influences its activity and the equilibrium of the entire ecosystem [1]. Decades of soil exploitation and intensive land management has led to its dramatic chemical degradation, especially in terms of nutrient losses [2]. Notwithstanding, in recent years, increasing awareness has been reversing the trend by the introduction of improved management technologies such as precision farming [3]. However, implementation of these practices requires that farmers are aware of within-field variations in soil properties [4]. A key soil property in soil management which reflects the state of the soil resource is the organic carbon content [5]. Soil total organic carbon (TOC) is usually defined as the total amount of the organic carbon-containing part in the soil, including residual components of original organic tissues, their degradation products and the products synthetized by soil fauna [6]. It represents a useful indicator of soil fertility as well as soil quality. It is also an important factor affecting long-term sustainability of agriculture, soil stability and crop yield [7]. Given the importance of TOC, there is a need for regular monitoring to detect changes in content and quality. However, most of the actual methods used to determine TOC are costly, time consuming and require specialized equipment, which is not always easily available for routine soil analysis by commercial or research laboratories [8]. TOC is usually determined by measuring the amount of organic carbon present using methods based on oxidation using gaseous oxygen in loss-on-ignition [9], elementary analysis methods or using strong and hazardous oxidants such as potassium dichromate [10]. Thus, soil monitoring using alternative methods is essential for the early detection of changes in TOC content and to provide intensive information about TOC content rapidly and at low cost, with an acceptable level of reliability. Near infrared (NIR) spectroscopy has well-known characteristics [11], and represents a radical break from conventional analytical assays, because a sample is characterized in terms of its whole light absorption properties rather than being treated with various chemicals to isolate specific components [12]. Moreover, due to the minimum sample preparation required as well as the possibility to be used directly in the field, the NIR approach has been particularly promising. To date, several studies have been published on NIR spectroscopy being applied to predict various soil properties in different configurations (especially in the laboratory, but also in the field using sampling, or on-the-go, using sensors embedded on a tractor) [13,14]. In addition, several soil properties have been predicted with acceptable accuracy, including for example soil organic matter [15], nitrogen [16], pH [17] and water content [18]. Recently, both NIR and medium infrared (MIR) regions of the spectra have been investigated for soil analysis. The NIR range is predominantly used for quantitative determination of soil parameters, whilst MIR is primarily useful for qualitative characterization of soil components [19].

Because soil is a complex combination of water, air, mineral and organic matter in various levels [20], the spectral response of soil is the result of the inherent absorption properties related to its chemical components and of scattering phenomena principally caused by its physical characteristics [21]. The relationship between reflectance and soil moisture [22], as well as the effects of particle size and soil surface geometry [23], have been widely studied in order to understand their influence on quantitative determination by NIR and their effects on prediction accuracy. Most of the cited NIR-based applications for TOC determination have provided spectra acquisition of air-dried, sieved or homogenized soil samples, assuming the need of transportation from field to lab and some sort of pretreatments before submitting samples to NIR analysis. The fact that field-portable NIR instruments are on the market or being developed, makes NIR spectroscopy (NIRS) particularly attractive for on-site measurements directly at field level, without any sample pretreatment.

Therefore, the aims of this study were to evaluate the effects of water content and soil particle size on NIR spectral signals of soil samples and on the quality of calibration, and to set up an NIR model for a fast and reliable prediction of soil TOC in order to develop a direct in-field measurement system based on NIR spectroscopy.

Moreover, the fact that field-portable NIR instruments are on the market or being developed, makes NIRS attractive for on-site or in situ measurements of C in soil.

## 2. Materials and Methods

### 2.1. Soil, Soil Sampling and Analysis

**Figure 1:**
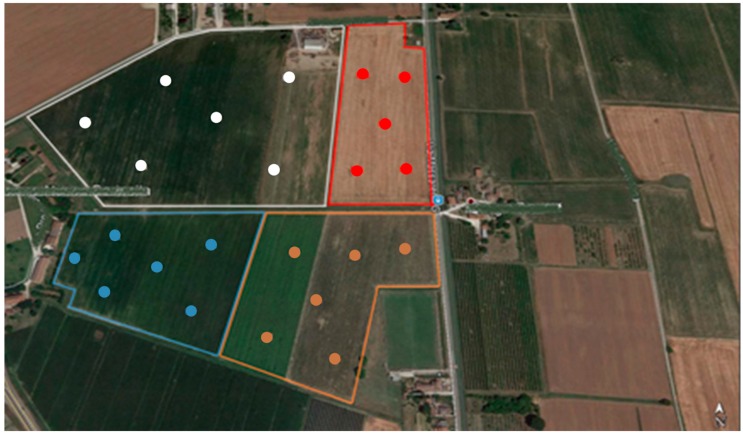
Sampling site area and sampling positioning (scale 1:200).

The study area was located in the Po-river valley, in the North-East of Italy. In May 2016, 23 topsoil samples (30 cm) and 23 subsoil samples (60 cm) were collected by means of a steel-coring bit. Soil in this area has the following composition: silt: 55%, sand: 27% and clay: 18%, with very little variation within the study site, as shown in previous studies on the same site. The sampling site had an overall surface of 190,000 m^2^, divided into four fields (Figure 1). Soil samples were collected through grid sampling (n = 23) from an area that had been previously studied with reference to nitrogen fertilization and presently under study for increasing carbon storage and soil organic matter [24]. An additional 16 samples, to be used as an external validation set, were collected within the same field in points different from the grid sampling and were randomly chosen from topsoil and subsoil.

Soil cores were broken apart by hand and in the laboratory, homogenised and put in a plastic bag while still moist, without any other treatment. All samples were then stored at −20°C until submitting to reference assays and spectra acquisition.

TOC was determined using a Carbon Analyzer TOC-V-CSM (Shimadzu, Tokio, Japan) after acidification with 2 M HCl to remove dissolved carbonate [25]. The instrument has a detection limit of 5 μg/L and a measurement accuracy expressed as CV 1.5%. All determinations were measured in triplicates to calculate reference data reproducibility.

### 2.2. Sample Treatments for NIR Spectral Acquisition

Three sets of NIR spectra were collected from the 46 soil samples: wet samples (WS), dried samples (DS) and ground and sieved samples (GSS), depending on the soil processing. NIR spectra of wet soil samples were collected by simply placing soil on glass Petri cups after being conditioned at room temperature for about 2 h to avoid interference with the spectra signals caused by low temperature (the WS set). The same samples were then dried overnight at 55 °C without any other manipulations and the spectra were registered the following day in the DS set. Finally, the dried soils were ground and passed through a 2 mm sieve to obtain a fine powder, and NIR spectra collected to obtain the GSS set.

### 2.3. NIR Spectroscopy

NIR spectra of soil samples were acquired with a NIRFLex N-500 (Büchi, Switzerland) set up with a polarization interferometer with TeO_2_ wedges and a Solids Cell Module (Büchi, Flawil, Switzerland), devised for housing standard 9.0 cm diameter Petri dishes (Schott, Mainz, Germany). Soil samples were loaded on the dishes and pressed by means of a stainless steel disk. Due to the eccentrically rotating cup housing, two scans for each sample were taken. The instrument was able to operate in the range of 5–35 °C, without any drift in the spectral signal. The reflectance spectra were collected in the 1000–2500 nm full-wavelengths interval using NIRWare 1.4 (Büchi, Flawil, Switzerland), at 2–4 scans/s. An optimized signal-to-noise ratio was guaranteed by averaging 64 scans for each spectrum, with an overall measurement time of 15 s. Internal and external references were acquired every 10 spectra in order to optimize the spectrum signal and set up the light source alignment.

### 2.4. NIR Statistics and Chemometrics

All chemometric analyses including calibration and validation were performed using NIRCal 5.4 (Buchi, Flawil, Switzerland). The raw optical data were pre-processed with full-multiplicative scatter correction (MSC), standard normal variate (SNV) and normalization by closure (NCL) as techniques to reduce the variability due to scattering, and 1st and 2nd derivatives (DV1 and DV2, respectively) to remove baseline offset and linear trends [26]. Depending on regression coefficients computed for reflectance at each wavelength, NIRCal 5.4 recommend the wavelengths interval for each data set. Principal component analysis (PCA) was carried out to perform discriminant qualitative principal component (PC) plots. Partial least squares regression (PLSR) was used as the regression model to correlate the reference data and the NIR predicted results. The optimal number of factors was assessed by calculating the predicted residual error sum of squares (PRESS) values, i.e., the sum of squares of deviation between predicted and reference values [27]. Cross-validation, or internal validation, was carried out as default software output, using the blockwise procedure, sharing out the calibration set into 3-fold blocks, and testing in turn one block as the validation set and the others as calibration sets. The software computed a series of calibration models and automatically selected the best one by comparing the squared Pearson correlation coefficient for both calibration (R^2^_cal_) and cross-validation (R^2^_CV_), standard error of calibration (SEC) and standard error of cross-validation (SECV). The regression model statistics were also evaluated in terms of relative prediction deviation (RPD), i.e., the relationship between the standard deviation (SD) of the entire population divided by the SEC [28]. Quality of calibration was formulated by Q-value, as a weighted combination of all relevant statistical measures (SEC, SEP, bias and regression coefficients). Q-value is automatically calculated by the software during the calibration protocol as:

$$\text{Q-value}=\frac{1}{1+\sum_{i=1}^{n}w_{i}v_{i}}$$

where *w* is the weight assigned for each statistical measure, *v* is the corresponding value of the statistical measures and *i* is the number of measures.

Q-value can be considered as an overall quality index, that classifies regressions by using a number between 0 (useless) and 1 (ideal). To be considered reliable, a calibration has to obtain a Q-value greater than 0.50 [29]. Mahalanobis distance criterion was used as the default method to find outliers in sample sets [30]. The standard error of the laboratory (SEL), i.e., the error of the reference data, was reported in order to benchmark the NIR statistics (SEC and SEP).

The calibrations for soils were then validated by means of external validation, and 16 new independent soil samples were collected in order to verify the predictive ability of NIR to obtain supplementary unknown soil samples. The accuracy of NIR-predicted data sets was measured as squared correlation coefficient (R^2^_EX.V_) and root mean standard error of prediction between predictions and reference values (RMSEP) [31]. NIR repeatability on predictions was calculated as the average of three acquisitions for each sample. Mean absolute error (MAE) with standard deviation (SD_MAE_) was also calculated to measure prediction accuracy [32].

Calibrations and validations were performed by means of a full-spectrum approach using all the wavelength in the interval of acquisition (1000–2500 nm).

## 3. Results and Discussion

### 3.1. Analysis of Spectral Data

**Figure 2:**
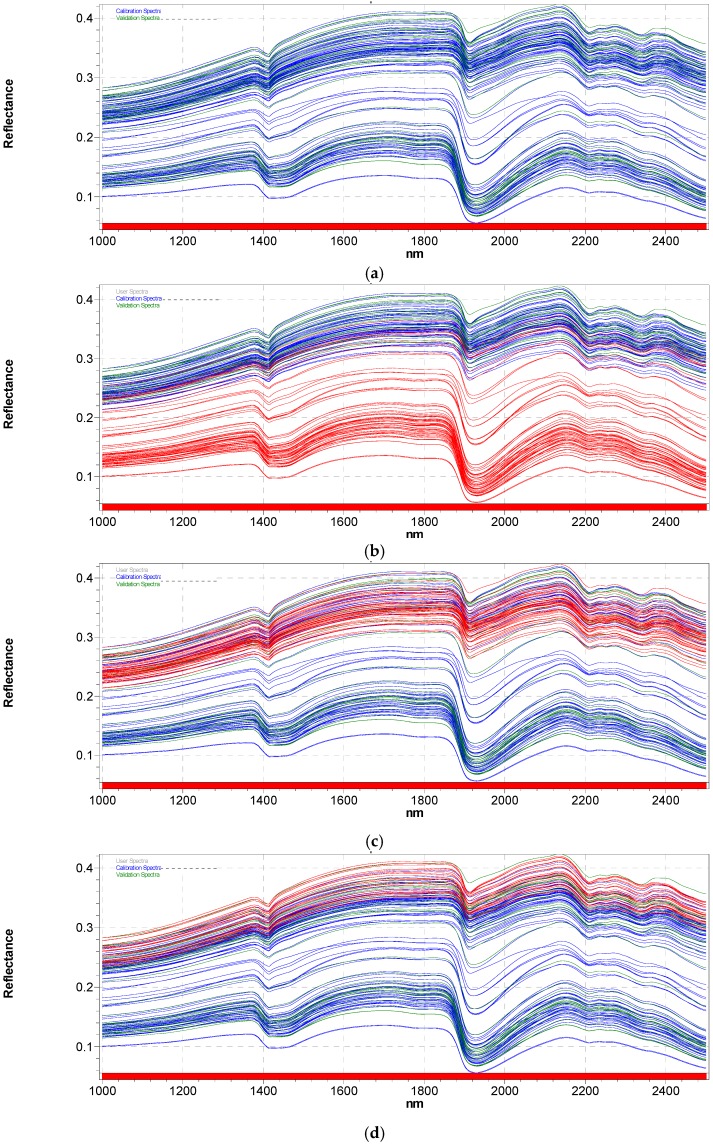
NIR absorbance spectra of (**a**) all soils sample sets; (**b**) the wet sample (WS) set highlighted in red; (**c**) the dried sample (DS) set highlighted in red; (**d**) the ground and sieved sample (GSS) set highlighted in red.

The diffuse reflectance NIR spectra of all soil samples are shown in Figure 2.

The major signals around 1400 nm (OH second overtone) and 1900 nm (OH third overtone), are caused by water absorption. Other signals related to organic constituents of soils are almost completely covered by the overwhelming presence of water, resulting in relatively smooth spectra. Various components of soil organic matter are generally more visible in the mid-infrared region, although the weak overtones and combination bands of fundamental vibrations occur in the near infrared range and can be exploited for analytical purposes [32]. As reported in the literature [33], absorption values below 1000 nm (not shown) are usually associated with humic compounds and with pigments derived from chlorophyll and phenolic compounds during decomposition of organic materials and plant residues. Even though often hidden, spectral signals at 1700 nm (CH_2_ overtones) and the range 2200–2300 nm (aliphatic CH and OH phenolic compounds) have been already correlated with soil characterization [34,35,36]. In particular, the region around 2200 nm was correlated with clay mineral [37], which is abundant in the soils we collected.

NIR reflectance of WS is strongly influenced by moisture, which induces an overall decrease in reflectance of soils with respect to dry samples [38]. As evidenced in Figure 2b, soil moisture markedly influences the soil scattering features and baseline offset effects. When soil moisture increases, soil particles absorb the water and then micro and macropores are filled with water. Depending on the moisture content, the water film around the soil particles determines a modification in the refractive index with respect to dry soils, where particles are surrounded by air [39], resulting in a larger part of light propagating deeper into the soil and consequently lowering the light scattering [40]. Moreover, it is worthwhile noting that the overall decrease in reflectance with increasing moisture content is not constant along with the spectrum, but it becomes more marked towards longer wavelengths, causing an increase in the slope of the spectral curves between 1800 and 2500 nm. Longer wavelengths are able to strongly adsorb water, emphasizing the change in reflectance [41]. This also generates a slight shift of the maximum of the peak at 1900 nm and peak broadening probably due to the increasing water content.

Conversely, particle size seemed not to have a significant effect on diffuse reflectance of soil samples. As expected, light scattering is more appreciable in the case of only dried samples because of the non-homogeneity of particles with respect to those that are ground and sieved, but comparing red spectra in Figure 2c,d, the overall spectral offsets are almost completely superimposed.

**Figure 3:**
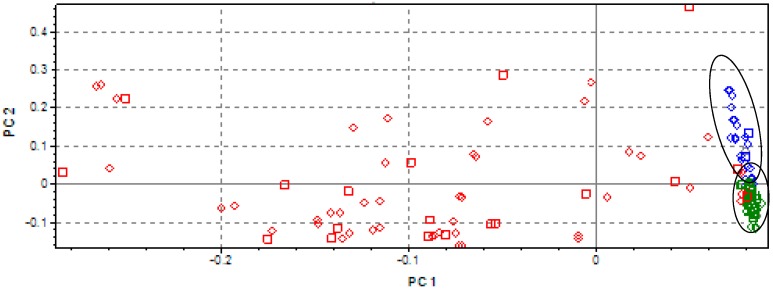
Principal component 2 (PC2) vs. PC1 plot for classification of soils samples based on Fourier transform-(FT) NIR spectra: WS (red); DS (green); GSS (blue).

The PCA carried out on all the original NIR spectra has confirmed what was detected by the visual inspection of the spectra (Figure 3).

Along the PC1, which explains 76.22% of the variance, samples are grouped based on moisture content, showing the greatest relevance of this variable. Equally interesting, inside the DS group, a separated sub-cluster corresponding to only dried (blue) and GSS (green) samples along PC2 (19.78% of variance explained) can be clearly recognized. Spectra were surely influenced by the homogeneity of particle size, but as a second-priority variable.

### 3.2. Spectra Pre-Treatments

**Figure 4:**
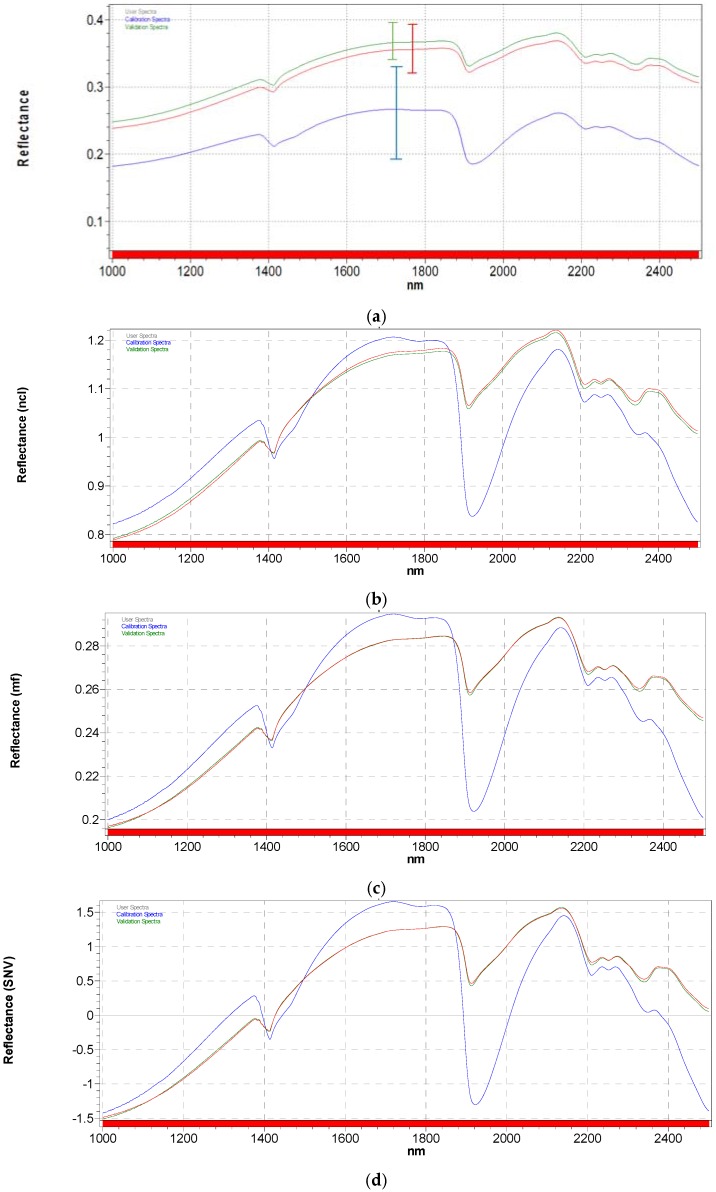
Pre-processing treatment on (**a**) average NIR spectra calculated separately from the WS (blue spectrum), DS (red spectrum) and GSS (green spectrum): effects of (**b**) normalization by closure (NCL); (**c**) multiplicative scatter correction (MSC); (**d**) standard normal variate (SNV).

As previously shown, the effect of light scattering is markedly predominant in our spectral set, generating spectral baseline shifts and non-linearity phenomena, which constitute the major part of the total variation of the signals. Spectra of soils usually contain noise and interferences, due to the fact that they are complex and multi-component matrices, that can be minimized applying mathematical treatments on the spectra before calibration [42]. Figure 4 shows the effects on average spectra calculated for the three sample sets (WS, DS and GSS) of NCL, MSC and SNV. They are the most widely used pre-processing techniques for NIR spectra to reduce the variability among samples due to scattering and adjust for baseline shifts [43].

In MSC, the light scattering is estimated and corrected for each sample relative to an ideal sample obtained by averaging the complete wavelength range of the data [44].

The signal correction concepts behind SNV and normalization are the same as those for MSC except that an average reference signal is not required. Instead, each observation is processed on its own, isolated from the reminder of the set [45]. As already discussed by Dhanoa et al. [46], there are some similarities among the three pretreatments up to a simple spectral rotation and offset correction (Figure 4b–d).

**Figure 5:**
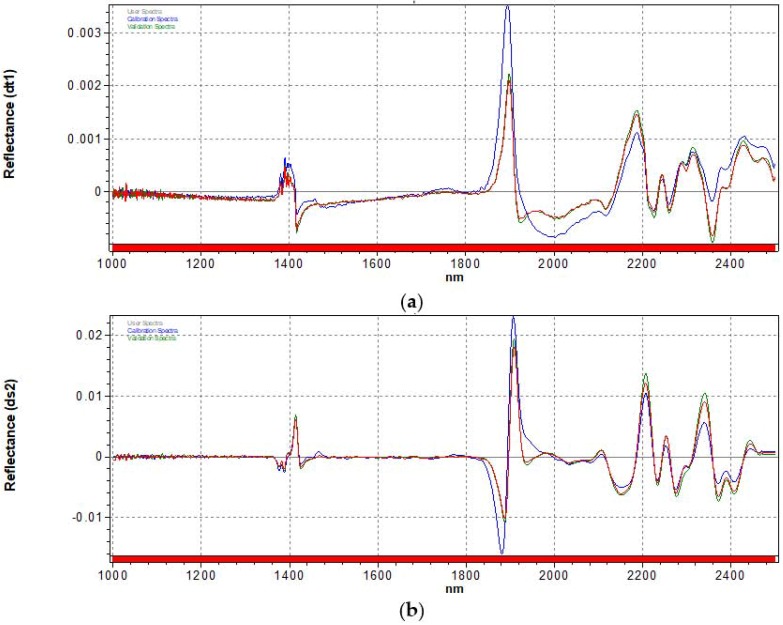
Pre-processing treatment on average NIR spectra calculated separately from the WS (blue spectrum), DS (red spectrum) and GSS (green spectrum): effects of (**a**) first derivative; (**b**) second derivative.

To reduce the effect of the background and remove the baseline shifts, Savitzky–Golay first and second derivative (5-point and 9-point segments, respectively) have also been applied to our spectra [47] (Figure 5).

It is worthwhile noting that in all cases, pretreatments are useful to reduce or eliminate the effect of undesired scattering due to particle size and shape as well as sample packing due to the different ground treatment of soils (red and green spectra in all figures), confirming that in that case the scattering phenomena are derived from the typical physical variations of the measured samples. When the particle size is larger than the wavelength, as is generally the case for solid samples analyzed by NIR, the anisotropic Lorenz–Mie [26] scattering is predominant and could be easily reduced or eliminated by means of the application of mathematical functions on the original spectra [48]. On the contrary, the presence of water in samples, besides light absorption, generates a Rayleigh scattering, or scattering by small particles as water molecules [49], which is strongly wavelength-dependent and nearly isotropic. The results of the application of preprocessing techniques are very different from the previous cases and more marked at larger wavelength [50].

### 3.3. PLSR Calibration Models for TOC Predictions

Using the reference assay values of TOC and the spectral data, original and pretreated, calibration models have been generated by the PLS regression method for DS (Table 1), GSS (Table 2) and WS (Table 3) sets. The reference analysis on soil samples gave values in the range of 0.99–2.42% of TOC, with a standard deviation of 0.33%. All the sample sets contained 46 samples, 30 as C-set and 16 as CV-set.

The reproducibility of reference data was 0.24%, whilst NIR measurement repeatability was in the range 0.11% for the dried and ground sample set to 0.14% for the WS set, slightly more than the expected value <0.5 SECV, surely due to the high heterogeneity of soils, which makes NIR determinations more subjected to errors [51].

**Figure 6:**
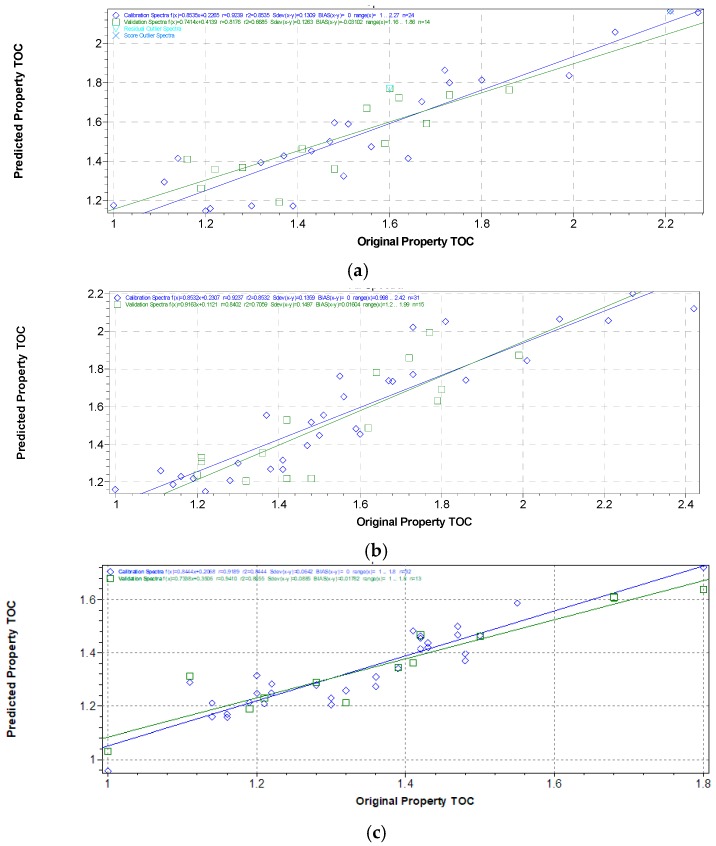
Calibration curves for parameter TOC in soils of original vs. predicted values from the WS dataset (**a**); DS dataset (**b**); GSS dataset (**c**).

The only outliers found in the case of DS and GSS sets were probably due to a failure in TOC determination for those samples, because they were recognized by the software as original property value-residual outliers. In the case of the WS set, the second outlier was recognized as a NIR-predicted value-residual outlier. Comparing the statistical results reported in the three tables, it is worthwhile noting that the DS and GSS sets show similar results and have overall better performances in terms of correlations and robustness of calibrations than the WS set, where the interference of water on spectra surely provides a decrease in the model’s efficiency. Different single pretreatments have generated different effects, SNV being the most suitable treatment for scattering correction, and DV2 (9 point) for smoothing. The combined use of SNV and DV2 have given the best calibration performances for all the three samples sets, also validated by the cross-validation results (Figure 6).

When compared with previous studies in estimating organic carbon content of soils by using visible/NIR spectra, the predictive performance obtained in this study was in accordance with Stevens et al. [5], on a large-scale EU soil survey of about 20,000 samples belonging to eight land-use types with R^2^ values from 0.76–0.96 and RPD values ranging from 1.74–2.88. Brown et al. [52] achieved a R^2^ value of 0.87 from a global scale. With 1011 soil samples, Shepherd and Walsh [53] obtained a R^2^ value of 0.80 for organic carbon content estimation. Moreover, Saiano et al. [54] estimated the soil carbon contents of 89 soils from a small and homogeneous area, Pantelleria Island, and achieved a considerably higher accuracy with a cross-validation R^2^ value of 0.951 and an RPD value of 4.49. Accordingly, Cozzolino et al. [33] reported a R^2^_CAL_ = 0.94–0.96 for silt and clay soils and R^2^_CAL_ = 0.89–0.92 for sand soils.

As reported by several Authors [55,56], moisture has dramatic effects on NIR reflectance, usually decreasing at increasing water content from dryness to saturation. In our case, we could confirm a general decreasing of spectral reflectance of wet samples compared with dry samples. However, the overall relatively low absolute water content of soil samples, which did not exceed 14% (w/w) and the narrow range of distribution, within six percentage points (analysis not shown), were probably due to the particular seasonal characteristics during sample collection, and resulted in an acceptable worsening of calibration parameters from GSS to WS models.

### 3.4. External Validation

After developing a calibration model, it is essential to evaluate the performance of the model with samples independent from those used to develop the model itself. External validation is a consolidated procedure that uses a separate data set to validate the calibrations before applying them in routine analysis where, especially in cases of complex and heterogeneous samples strongly affected by composition and structure, cross validation alone is not sufficiently reliable to trust model performances. Accordingly, another 16 independent soil samples, randomly chosen between topsoil and subsoil samples, were collected as previously described, and submitted to NIR detection and to TOC reference assays. The results of these supplementary tests have been reported in terms of NIR-predicted TOC (Table 4) against the original property results. The reference TOC values were in the range of 1.05–2.21% with a standard deviation of 0.32%.

The three sets of NIR-predicted data were evaluated by one-way ANOVA and were not found to be significantly different (*p* < 0.05) [57]. As a consequence, both particle size and moisture seem not to dramatically influence the predictions, or not in a way that can be taken into account at the moment of sample collection. Samples could be collected and spectra acquired without any physical pre-treatments such as drying or grinding, but nevertheless still obtaining reliable NIR predictions for samples with different physical characteristics.

**Figure 7:**
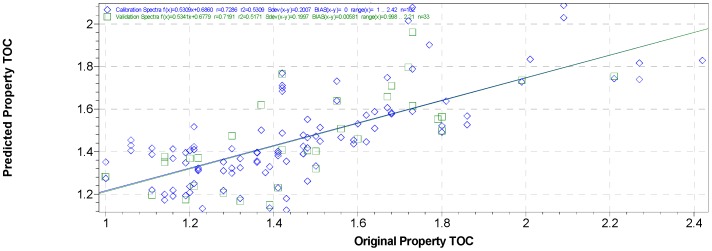
Calibration curve for parameter TOC in soils of original vs. predicted values grouping together all three data sets.

As a consequence, an attempt to gather all samples together in a single set and a unique recalculated calibration model, was carried out. The regression model (three factors), and the cross-validation curve are shown in Figure 7.

In this way, it was possible to include within a unique calibration all the signal variations derived from both water and particle size, that generate scattering on the spectra. A total of 138 spectra have been processed with SNV and 2nd derivative as pretreatments and used to develop the calibration model. Blockwise cross-validation assigned 92 in the C-set and 46 in the V-set. The regression coefficient (R^2^) and the standard error of calibration (SEC) were 0.78 and 0.17, respectively. The validation samples were predicted with a SECV of 0.18 and a R^2^ of 0.80. Based on the DW statistics, both the C-set and V-set showed no autocorrelation. RPD for calibration and cross-validation were 1.9 and 1.8, respectively, which are satisfactory in terms of the predictive ability of the model.

**Figure 8:**
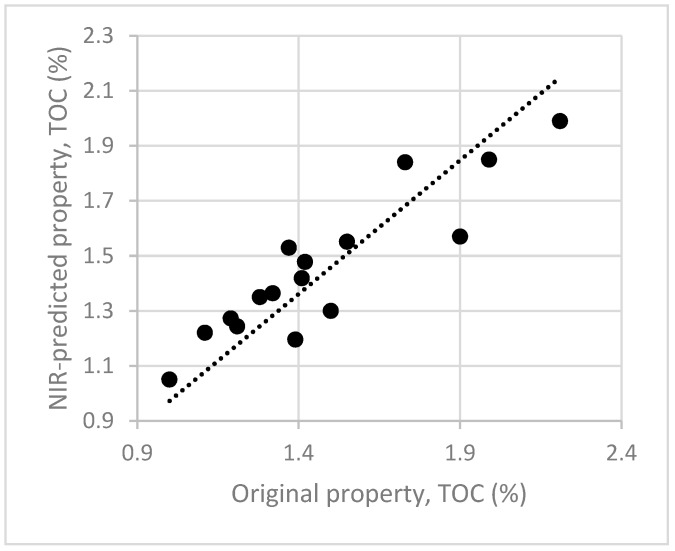
External validation of the NIR calibration model for the prediction of TOC content in unknown samples of soils.

The new calibration model was validated using the same 16 samples previously collected, with R^2^ of 0.71 and RMSEP of 0.30 (Figure 8).

## 4. Conclusions

NIR spectroscopy has demonstrated great potential to predict TOC in soil samples with different characteristics in terms of particle size and moisture content. A lab-scale method to predict TOC has been set up and proposed for soils at different moisture content and two particle sizes. Results have shown linearity and satisfactory regression models in all the three cases separately. The absence of differences in the prediction capability of independent samples has demonstrated that the general effects of physical soil characteristics do not generate dramatic interferences with spectral signals and TOC quantifications. Despite a slight worsening of the prediction capacity, the possibility to gather all samples and build a unique calibration model has permitted us to encompass the two principal sources of spectral offsets and shifts in the calibration model, increasing its robustness and reliability with unknown samples. Future improvements of this application could permit performing NIR analysis of soils directly in field by potentially using a probe connected to the NIR instrument.

## Figures and Tables

**Table 1:** The optimal partial least squares (PLS) model prediction results for total organic carbon (TOC) and the corresponding statistical parameters of the various single and combined pretreatments for the DS set (NCL = normalization by closure; MSC = full-multiplicative scatter correction, SNV = standard normal variate; DV1 = 1st derivative; DV2 = 2nd derivative).

Pre-Treatment Applied	Outliers	F	R^2^_CAL_	SEC	R^2^_CV_	SECV	RPD_CAL_	RPD_CV_	DW	Q-Value
NCL	1	5	0.91	0.13	0.88	0.15	2.50	2.15	1.6	0.76
MSC	1	4	0.91	0.14	0.91	0.14	2.43	2.31	1.4	0.69
SNV	1	6	0.92	0.13	0.92	0.15	2.64	2.23	1.5	0.68
DV1	1	6	0.82	0.19	0.76	0.16	0.34	2.04	1.9	0.52
DV2	1	3	0.92	0.15	0.92	0.18	2.25	1.89	1.8	0.88
SNV + DV1	1	4	0.91	0.11	0.91	0.13	3.04	2.48	1.6	0.71
SNV + DV2	1	3	0.96	0.06	0.93	0.09	5.31	3.52	2.0	0.89

**Table 2:** The optimal PLS model prediction results for TOC and the corresponding statistical parameters of the various single and combined pretreatments for the GSS set. (NCL = normalization by closure; MSC = full-multiplicative scatter correction, SNV = standard normal variate; DV1 = 1st derivative; DV2 = 2nd derivative).

Pre-Treatment Applied	Outliers	F	R^2^_CAL_	SEC	R^2^_CV_	SECV	RPD_CAL_	RPD_CV_	DW	Q-Value
NCL	1	7	0.89	0.12	0.74	0.16	2.81	2.12	0.7	2.32
MSC	1	2	0.90	0.11	0.89	0.14	3.16	2.34	0.5	2.45
SNV	1	3	0.95	0.11	0.93	0.10	3.13	3.28	0.7	1.91
DV1	1	4	0.90	0.10	0.87	0.15	3.38	2.21	0.7	1.87
DV2	1	2	0.96	0.09	0.92	0.10	3.45	3.25	0.8	2.54
SNV + DV1	1	2	0.92	0.10	0.90	0.11	3.28	3.01	0.7	2.18
SNV + DV2	1	2	0.99	0.09	0.93	0.10	3.52	3.31	0.8	2.64

**Table 3:** The optimal PLS model prediction results for TOC and the corresponding statistical parameters of the various single and combined pretreatments for the WS set. (NCL = normalization by closure; MSC = full-multiplicative scatter correction, SNV = standard normal variate; DV1 = 1st derivative; DV2 = 2nd derivative).

Pre-Treatment Applied	Outliers	F	R^2^_CAL_	SEC	R^2^_CV_	SECV	RPD_CAL_	RPD_CV_	DW	Q-Value
NCL	2	3	0.79	0.16	0.73	0.23	2.13	1.53	0.5	1.21
MSC	2	5	0.74	0.19	0.69	0.17	1.70	1.93	0.5	1.31
SNV	2	3	0.80	0.16	0.78	0.21	2.07	1.57	0.5	1.62
DV1	1	4	0.74	0.14	0.74	0.17	2.31	1.93	0.6	1.65
DV2	1	4	0.89	0.13	0.85	0.18	2.50	1.90	0.6	1.58
SNV + DV1	2	4	0.93	0.08	0.91	0.10	4.03	3.64	0.6	1.83
SNV + DV2	1	3	0.96	0.11	0.92	0.12	3.07	2.81	0.7	2.01

**Table 4:** External validation of the NIR calibration model for the prediction of TOC in unknown samples of WS, DS and GSS sets. Range and SD refer to NIR predicted values.

Sample Set	Range	SD	MAE ± SD_MAE_	Outliers	R^2^_EX.VAL._	RMSEP	Bias	Slope
WS	1.21%–2.05%	0.32%	0.11 ± 0.08	0	0.76	0.25	0.38	0.72
DS	1.16%–1.99%	0.33%	0.10 ± 0.09	0	0.79	0.27	0.21	0.87
GSS	1.00%–2.16%	0.27%	0.13 ± 0.08	0	0.83	0.26	0.20	0.88

## References

[1] Curry J.P., Good J.A. (1992). Soil faunal degradation and restoration. Adv. Soil Sci..

[2] Virto I., Imaz M.J., Fernández-Ugalde O., Gartzia-Bengoetxea N., Enrique A., Bescansa P. (2015). Soil Degradation and Soil Quality in Western Europe: Current Situation and Future Perspectives. Sustainability.

[3] Keskin M., Sekerli Y.E. (2016). Awareness and adoption of precision agriculture in the Cukurova region of Turkey. Agron. Res..

[4] McBratney A., Whelan B., Ancev T., Bouma J. (2015). Future directions of precision agriculture. Precis. Agric..

[5] Stevens A., Nocita M., Tóth G., Montanarella L., van Wesemael B. (2013). Prediction of soil organic carbon at the European scale by visible and near infrared reflectance spectroscopy. PLoS ONE.

[6] Kogel-Knabner I. (2002). The macromolecular organic composition of plant and microbial residue as inputs to soil organic matter. Soil Biol. Biochem..

[7] Loveland P., Webb J. (2003). Is there a critical level of organic matter in the agricultural soils of temperate regions: A review. Soil Till. Res..

[8] Schumacher B.A. (2002). Methods for the Determination of Total Organic Carbon (TOC) in Soils and Sediments.

[9] Chatterjee A., Lal R., Wielopolski L., Martin M.Z., Ebinger M.H. (2009). Evaluation of different soil carbon determination methods. Crit. Rev. Plant Sci..

[10] Jimenez R.R., Ladha J.K. (1993). Automated elemental analysis: A rapid and reliable but expensive measurement of total carbon and nitrogen in plant and soil samples. Commun. Soil Sci. Plant Anal..

[11] Tamburini E., Marchetti M.G., Pedrini P. (2014). Monitoring key parameters in bioprocesses using near-infrared technology. Sensors.

[12] Siesler H.W., Ozaki Y., Kawata S., Heise H.M. (2008). Near-Infrared Spectroscopy: Principles, Instruments, Applications.

[13] Schellberg J., Hill M.J., Gerhards R., Rothmund M., Braun M. (2008). Precision agriculture on grassland: Applications, perspectives and constraints. Eur. J. Agron..

[14] Shi Z., Huang J., Li S. (2016). Correction: In Situ Measurement of Some Soil Properties in Paddy Soil Using Visible and Near-Infrared Spectroscopy. PLoS ONE.

[15] Luce M.S., Ziadi N., Zebarth B.J., Grant C.A., Tremblay G.F., Gregorich E.G. (2014). Rapid determination of soil organic matter quality indicators using visible near infrared reflectance spectroscopy. Geoderma.

[16] Shi T., Cui L., Wang J., Fei T., Chen Y., Wu G. (2013). Comparison of multivariate methods for estimating soil total nitrogen with visible/near-infrared spectroscopy. Plant Soil.

[17] Wang Y., Huang T., Liu J., Lin Z., Li S., Wang R., Ge Y. (2015). Soil pH value, organic matter and macronutrients contents prediction using optical diffuse reflectance spectroscopy. Comput. Electron. Agric..

[18] Dalal R.C., Henry R.J. (1986). Simultaneous determination of moisture, organic carbon, and total nitrogen by near infrared reflectance spectrophotometry. Soil Sci. Soc. Am. J..

[19] Gehl R.J., Rice C.W. (2007). Emerging technologies for in situ measurement of soil carbon. Clim. Chang..

[20] Beyer L. (1996). The chemical composition of soil organic matter in classical humic compound fractions and in bulk samples—A review. J. Soil Sci. Plant Nutr..

[21] Gholizadeh A., Borůvka L., Saberioon M., Vašát R. (2013). Visible, near-infrared, and mid-infrared spectroscopy applications for soil assessment with emphasis on soil organic matter content and quality: State-of-the-art and key issues. Appl. Spectrosc..

[22] Weidong L., Baret F., Xingfa G., Qingxi T., Lanfen Z., Bing Z. (2002). Relating soil surface moisture to reflectance. Remote Sens. Environ..

[23] Martens H., Nielsen J.P., Engelsen S.B. (2003). Light scattering and light absorbance separated by extended multiplicative signal correction. Application to near-infrared transmission analysis of powder mixtures. Anal. Chem..

[24] Castaldelli G., Colombani N., Vincenzi F., Mastrocicco M. (2013). Linking dissolved organic carbon, acetate and denitrification in agricultural soil. Environ. Earth Sci..

[25] Potter B.B., Wimsatt J.C. (2005). Method 415.3. Determination of Total Organic Carbon and Specific UV Absorbance at 254 nm in Source Water and Drinking Water.

[26] Rinnan A., van den Berg F., Engelsen B.S. (2009). Review of the most common pre-processing techniques for near-infrared spectra. Trends Anal. Chem..

[27] Martens H., Naes T. (1988). Multivariate Calibration.

[28] Williams P.C. (2001). Near-Infrared Technology in the Agricultural and Food Industries.

[29] Durbin J., Watson G.S. (1950). Testing for serial correlation in least squares regression. Biometrika.

[30] Rocke D.M., Woodruff D.L. (1996). Identification of Outliers in Multivariate Data. J. Am. Stat. Assoc..

[31] Dardenne P. (2010). Some Considerations about NIR Spectroscopy: Closing Speech at NIR-2009. NIR News.

[32] Reeves J.B., McCarty G.W., Reeves V.B. (2001). Mid-infrared and diffuse reflectance spectroscopy for the quantitative analysis of agricultural soils. J. Agric. Food Chem..

[33] Cozzolino D., Moron A. (2006). Potential of near-infrared reflectance spectroscopy and chemometrics to predict soil organic carbon fractions. Soil Till. Res..

[34] Fidencio P.H., Poppi R.J., De Andrade J.C., Cantarella H. (2002). Determination of organic matter in soil using near-infrared spectroscopy and partial least squares regression. Commun. Soil Sci. Plant Anal..

[35] Stenberg B., Rossel R.A.V., Mouazen A.M., Wetterlind J. (2010). Chapter five-visible and near infrared spectroscopy in soil science. Adv. Agron..

[36] Viscarra Rossel R.A., Walvoort D.J.J., McBratney A.B., Janik L.J., Skjemstad J.O. (2006). Visible, near-infrared, mid-infrared or combined diffuse reflectance spectroscopy for simultaneous assessment of various soil properties. Geoderma.

[37] Daughtry C.S.T. (2001). Discriminating crop residues from soil by shortwave infrared reflectance. Agron. J..

[38] Stoner E.R., Baumgardner M.F. (1981). Characteristic variations in reflectance of surface soils. Soil Sci. Soc. Am. J..

[39] Bach H., Mauser W. Modelling and model verification of the spectral reflectance of soils under varying moisture conditions. Proceedings of the IEEE Geoscience and Remote Sensing Symposium, IGARSS ’94.

[40] Somers B., Gysels W., Verstraeten W.W., Delalieux S., Coppin P. (2010). Modeling moisture-induced soil reflectance changes in cultivated sandy soils: A case study in citrus orchards. Eur. J. Soil Sci..

[41] Lobell D., Asner G. (2002). Moisture effects on soil reflectance. Soil Sci. Soc. Am. J..

[42] Chen H., Song Q., Tang G., Feng Q., Lin L. (2013). The combined optimization of Savitzky-Golay smoothing and multiplicative scatter correction for FT-NIR PLS models. ISRN Spectrosc..

[43] Bazar G., Kovacs Z., Tsenkova R. (2016). Evaluating spectral signals to identify spectral error. PLoS ONE.

[44] Blanco M., Coello J., Montoliu I., Romero M.A. (2001). Orthogonal signal correction in near infrared calibration. Anal. Chim. Acta.

[45] Barnes R.J., Dhanoa M.S., Lister S.J. (1989). Standard normal variate transformation and de-trending of near-infrared diffuse reflectance spectra. Appl. Spectrosc..

[46] Dhanoa M.S., Lister S.J., Sanderson R., Barnes R.J. (1994). The link between multiplicative scatter correction (MSC) and standard normal variate (SNV) transformations of NIR spectra. JNIRS.

[47] Czarnecki M.A. (2015). Resolution enhancement in second-derivative spectra. Appl. Spectrosc..

[48] Pasquini C. (2002). Near infrared spectroscopy: Fundamentals, practical aspects and analytical applications. J. Braz. Chem. Soc..

[49] Jacques S.L. (2013). Optical properties of biological tissues: A review. Phys. Med. Biol..

[50] Carter G.A. (1991). Primary and Secondary Effects of Water Content on the Spectral Reflectance of Leaves. Am. J. Bot..

[51] Salguero-Chaparro L., Baeten V., Abbas O., Peña-Rodríguez F. (2012). On-line analysis of intact olive fruits by vis–NIR spectroscopy: Optimisation of the acquisition parameters. J. Food Eng..

[52] Brown D.J., Shepherd K.D., Walsh M.G., Dewayne Mays M., Reinsch T.G. (2006). Global soil characterization with VNIR diffuse reflectance spectroscopy. Geoderma.

[53] Shepherd K.D., Walsh M.G. (2002). Development of reflectance spectral libraries for characterization of soil properties. Soil Sci. Soc. Am. J..

[54] Saiano F., Oddo G., Scalenghe R., La Mantia T., Ajmone-Marsan F. (2013). DRIFTS sensor: Soil carbon validation at large scale (Pantelleria, Italy). Sensors.

[55] Minasny B., McBratney A.B., Bellon-Maurel V., Roger J.M., Gobrecht A., Ferrand L., Joalland S. (2011). Removing the effect of soil moisture from NIR diffuse reflectance spectra for the prediction of soil organic carbon. Geoderma.

[56] Nocita M., Stevens A., Noon C., van Wesemael B. (2013). Prediction of soil organic carbon for different levels of soil moisture using Vis-NIR spectroscopy. Geoderma.

[57] Górecki T., Smaga Ł. (2015). A comparison of tests for the one-way ANOVA problem for functional data. Computat. Stat..

